# Radiobiological Modeling with Monte Carlo Tools – Simulating Cellular Responses to Ionizing Radiation

**DOI:** 10.1177/15330338251350909

**Published:** 2025-07-17

**Authors:** Tiago André Azevedo, Ana Margarida Abrantes, João Carvalho

**Affiliations:** 1 426090 CFisUC, Department of Physics, University of Coimbra, Coimbra, Portugal; 2 ICBR-Coimbra Institute for Clinical and Biomedical Research – Area of Environment, Genetics and Oncobiology ( 646815 CIMAGO), Faculty of Medicine, Institute of Biophysics, University of Coimbra, Coimbra, Portugal

**Keywords:** radiobiology, cancer hallmarks, *in silico* modeling, Monte Carlo tools, biomathematical model

## Abstract

As the prevalence of cancer continues to rise in a rapidly aging population, the integration of advancements in computational capabilities with oncological practices presents promising opportunities for enhancing cancer treatment management. *In silico* modeling has emerged as a key approach for studying the radiobiological aspects of cancer, providing novel pathways for understanding cellular mechanisms and potential future improvements in clinical radiotherapy. This review examines significant advancements and ongoing challenges in simulating the complex interactions of ionizing radiation with cancer cells. We explore the utility and limitations of current *in silico* models, including agent-based models and hybrid approaches that integrate cellular behavior with radiobiological effects using Monte Carlo tools. The paper highlights key developments that have enabled more accurate simulations of DNA damage, various repair processes, and the influence of the microenvironment on cellular radiosensitivity. Looking ahead, we address the need for further refinement of these models and their integration with experimental data to enhance predictive accuracy and potential clinical applications. The capacity of these models to potentiate personalized cancer therapy is emphasized, highlighting the ongoing shift towards more comprehensive and sophisticated computational approaches.

## Introduction

Cancer encompasses a variety of diseases that share several characteristics and globally remains a leading cause of death, especially as populations age.^1,2^ Despite significant advances in cancer treatment technologies that have improved survival rates,^3^ challenges persist, particularly related to treatment with radiation, where physical, chemical, and biological processes impacting treatment are often not fully accounted for. This gap underscores the potential of *in silico* modeling to improve treatment approaches by simulating the complex biological interactions at play.

Radiobiology is the study of the effects of ionizing radiation interaction with living matter.^4^ The strong evidence that DNA is the critical target for most biological effects of ionizing radiation,^5^ has shown that *in silico* simulations can be a valuable tool when combined in an experimental setup, with applications that range from accurately depicting the 3D dose distribution of radiation^6^ to simulating DNA damage and repair. This article divides the discussion on *in silico* modeling into three interconnected stages: the growth and reorganization of cancer cells, the interaction of radiation with cellular components, and the simulation of radiation effects, including DNA repair and cellular response mechanisms. By exploring these stages, this review aims to detail how computational models can integrate and improve the understanding and efficacy of radiotherapy.

## Fundamentals

The linear-quadratic (LQ) model forms the cornerstone of radiobiological studies, providing a framework to understand the probability of cell survival under radiation exposure. The model is succinctly expressed by the equation:

$$S(D)=e^{-(\alpha D+\beta D^{2})}$$

where S(D) represents the survival fraction at a given dose *D*, with α and β being fitting parameters. This dual-action framework (wherein damage increases linearly and quadratically with dose) was first introduced to accommodate observed cell-survival curves.^7,8^ Despite its simplicity, this model provides an adequate approximation for the range of doses typically used in clinical radiotherapy.^4^ The LQ model has gained widespread acceptance in radiotherapy practice due to its robust validation through experimental and clinical data, and is frequently employed to solve practical clinical issues, such as compensating for missed treatment days, comparing different treatment schemes, and designing novel treatment schedules for clinical trials.^9^ However, the model's limitations become apparent in scenarios where biological responses to radiation are influenced by complex cellular interactions. For example, the LQ model expects an instantaneous delivery of the dose and does not take into account the repair of sub-lethal damage that occurs when the dose is given slowly.^10^ Furthermore, the LQ model overestimates radiation-mediated killing at high doses because it predicts a continuous downward bend (due to the quadratic portion), whereas the dose-response data shows a linear effect at doses above 12 Gy.^11,12^

DNA carries the basic genetic information that controls cellular function, and, as such, the damage and the type of damage done to this molecule plays a leading role in radiobiological effects.^13^ Radiation-induced DNA damage is typically divided into single-strand breaks (SSBs) and more severe double-strand breaks (DSBs), the latter involving simultaneous breaks in both DNA strands.^14^ Double-strand breaks are recognized as the most important lesions caused by ionizing radiation, as they are highly correlated with cell viability decrease.^5^^(p202)^ Unlike SSBs, where the undamaged complementary strand can serve as a template for accurate repair, DSBs often require more complex and error-prone repair mechanisms. The repair of DSBs varies with the cell cycle phase. During the S and G2 phases, when DNA replication occurs, homologous recombination (HR) can utilize the sister chromatid for an error-free repair process. In contrast, non-homologous end joining (NHEJ) and the less precise microhomology-mediated end-joining (MMEJ) are repair mechanisms active throughout the cell cycle, but do not guarantee that the original DNA sequence is restored.^15^ Such error-prone repairs can lead to genomic instability, resulting in cytotoxicity and mutation propagation,^16^ which are critical concerns in cancer development and treatment.

The particles that interact with biological matter with sufficient energy as to cause chemical or biological damage to the DNA molecule can be classified as being directly or indirectly ionizing. The former applies to charged particles that will experience Coulomb or even nuclear interactions, while the latter applies to uncharged particles that deposit energy indirectly, through secondary particles. A key metric for evaluating the impact of these interactions on biological tissues is the Linear Energy Transfer (LET), defined as the average energy, ⟨dE⟩ , deposited by a charged particle per unit distance, dx . Particles with high LET, such as alpha particles and protons, are highly ionizing and cause significant biological damage by producing a higher frequency of DSBs and complex DNA damage.^17,18^ In contrast, lower LET radiation, like x-rays and beta particles, typically used in clinical settings, causes less direct damage but can still lead to significant biological effects through interactions with the cellular environment.

Water, as the most abundant molecule in cells, plays a central role in the interactions of ionizing radiation (IR) with biological matter.^17,19^ The radiation primarily causes water radiolysis, a process where water molecules are ionized and decomposed, generating highly reactive short-lived radicals like hydroxyl ( $OH^{\cdot }$ ) and hydrogen ( $H^{\cdot }$ ). These radicals are particularly reactive towards cellular molecules, including DNA, lipids, and other subcellular constituents, leading to significant cellular damage.^20^ In hypoxic conditions, the instability of these radicals limits the extent of the radiolytic reactions, allowing some repair mechanisms to restore damaged DNA. However, the presence of oxygen dramatically changes this outcome. Oxygen interacts with the radicals to form additional reactive oxygen species (ROS), such as the superoxide and hydroxyl radical, which further increases the concentration of DNA-damaging agents.^21^ Moreover, oxygen reacts with the fractured DNA strands to create stable peroxides, a process known as oxygen fixation effect, which inhibits the repair of DNA in oxygen-rich environments.^22^ This leads to the phenomenon known as oxygen enhancement ratio (OER), where the presence of oxygen amplifies the extent of radiation-induced damage. The OER is particularly significant for low LET radiation like gamma-rays, where the presence of oxygen is crucial to maximize its indirect effects. This mechanism underscores the importance of oxygen in effective tumor control, and the OER can be exploited for enhanced treatment efficacy.

## Radiobiological Hallmarks of Cancer

Cancer cells are fundamentally different from their normal counterparts, exhibiting a range of genetic alterations that enable their survival under adverse conditions and present unique challenges and opportunities for treatment approaches like radiotherapy. These alterations include evasion of growth suppression, sustained proliferative signaling, resistance to cell death, induction of angiogenesis, achievement of replicative immortality, and activation of invasion and metastasis.^23^ These are the hallmarks of cancer outlined by Hanahan and Weinberg, which has been the basis in the development of treatment strategies.^24^

In radiotherapy, these biological insights are strategically exploited through an approach known as the 5Rs, where each R targets a specific aspect of tumor biology: radiosensitivity, repair, redistribution, repopulation, and reoxygenation. Recent advances have also introduced a sixth R—reactivation of the immune system. This approach enhances the therapeutic efficacy of radiotherapy by engaging the body's natural defenses, providing an additional layer to the cancer fight strategy.^25^

Radiosensitivity varies significantly throughout the cell cycle, influenced by both the phase and the cell phenotype.^26^ Specifically, cells in the late S-Phase exhibit the highest resistance to radiation, whereas those in the G2/M phase are markedly more sensitive.^20^ This increased sensitivity is due, in part, to chromatic condensation during the G2/M phase, which compacts the DNA structure and makes it more susceptible to damage from ionizing radiation. Moreover, DNA repair mechanisms are less active in this phase compared to the S and G1 phases, granting less time for the cell to detect and correct radiation-induced damage.^27^ Following irradiation, many cancer cells are temporarily arrested in the G2-M phase, a process known as synchronization. This allows for targeted treatments when cells are most vulnerable. As the cell cycle progresses, cells shift from less sensitive phases to those where they are more susceptible to radiation.

Managing repopulation effectively is crucial in fractionated radiotherapy, where the aim extends beyond merely damaging cancer cells to strategically timing the doses to prevent rapid tumor regrowth between sessions. This timing controls growth and maximizes the therapeutic outcome. Additionally, fractionation facilitates the re-oxygenation of cancer cells, addressing the imbalance between oxygen demand and supply caused by tumor's rapid proliferation.^28^ This often leads to intermittent hypoxia due to fluctuations in blood perfusion or persistent limitations in oxygen diffusion.^29^ Tumor hypoxia significantly contributes to resistance in low-LET radiation treatments, making its management a key point in therapy planning.^30^ Post-irradiation, the selectively eliminated well-oxygenated cells lead to a temporarily increased proportion of hypoxic cells within the tumor. These surviving hypoxic cells subsequently undergo reoxygenation, initiating a new equilibrium between oxygenated and hypoxic cells. This cyclic process, enhanced by each radiation session, gradually reduces the overall number of hypoxic cells, progressively diminishing one of the major barriers to effective radiotherapy.^31^ By understanding and manipulating these dynamics, clinicians can tailor fractionation schedules to optimize tumor oxygenation, ultimately improving treatment efficacy and patient outcome.

After exploring the complex radiobiological hallmarks of cancer, we recognize the intricate interplay between cellular mechanisms, such as DNA damage, repair processes, and radiation effects across various cell cycle stages. These biological complexities pose significant challenges in predicting therapeutic outcomes and optimizing radiotherapy protocols. Given the inherent variability in tumor responses, there is a critical need for tools capable of simulating these diverse biological reactions under different therapeutic conditions. In this context, *in silico* modeling techniques can play a significant role. These computational packages are powerful tools for capturing the multifaceted nature of cancer biology, enabling researchers and clinicians to predict with greater accuracy the efficacy of various radiotherapy techniques. Although *in silico* models simulate interactions at the cellular and molecular levels, their utility is currently limited by the availability of biological-level data at patient resolution, which is essential for truly personalized care. Nevertheless, these models enhance fundamental radiobiological understanding and contribute to the refinement of clinical protocols. In the following sections, we explore specific *in silico* models in greater detail, illustrating their potential impact on radiotherapy treatment planning, patient outcomes and as drivers to further research.

## *In Silico* Modelling Techniques

Building on our foundational knowledge of radiobiological hallmarks in cancer, this section emphasizes coupled model frameworks that translate complex biological interactions into predictive tools. By examining these techniques, we deepen our understanding of how theoretical models correlate with practical therapeutic strategies. The simulation process of the proposed coupled model framework involves two main stages: initially focusing on tumor growth and evolution—highlighting crucial interactions within the cellular microenvironment such as oxygen and nutrients. Subsequently, tumor irradiation is simulated, accounting for both direct and indirect radiation effects, including water radiolysis and oxygen level impact. Post-irradiation, models integrate DNA repair mechanisms before advancing to further cellular growth. This section looks into specific modeling techniques employed in radiobiological research, with each component discussed in detail. These examinations are not only supported by specific examples but are also framed within the general categorization system developed by J.A. Bull and H.M. Byrne.^32^ Their framework outlines six key mathematical hallmarks that guide the complexity and application of computational models in oncology. These hallmarks are useful in developing sophisticated cancer models and include distinctions between single and hybrid frameworks, and homogeneous versus heterogeneous systems, among others.

### ODE Models

Ordinary differential equation (ODE) models, within the deterministic frameworks outlined by Bull and Byrne, provide a computationally efficient method to simulate cancer progression and treatment. These models use predefined, non-stochastic and usually non-linear equations to predict cancer behavior under controlled variables, which are crucial for theoretical research. Particularly, ODEs are very helpful in illustrating fundamental biological dynamics such as exponential growth and carrying capacities under environmental constraints. For instance, exponential models effectively describe unchecked tumor growth in early stages, while logistic models are better at representing the growth plateau due to microenvironmental limitations. Each model is adapted to different tumor stages and cell types, illustrating the versatile applications of ODEs in oncology research. Nevertheless, the preference for ODEs often stems from their analytical simplicity rather than their empirical accuracy^33^ which can limit their applicability in scenarios requiring complex biological fidelity

### Agent-Based Models

While ODE models provide a robust framework for understanding general trends in tumor growth and treatment response, they often fall short in capturing the complex, stochastic nature of tumor dynamics and the spatial heterogeneity inherent to the tumor microenvironment. Agent-based models (ABMs) address these complexities by simulating the individual behaviors and interactions of cells within a tumor. Each agent in these models represents a cell or a group of cells behaving based on localized information and rules, allowing for the detailed study of emergent behaviors, spatial relationships, and the effects of individual cellular actions and properties.^34^ ABMs excel in modeling complex interactions not just between cells, but also between cells and their environment, capturing the stochastic behaviors of individual cells within a tumor and facilitating the simulation of differential spatial-temporal evolution and inter and intra tumor heterogeneity. The simulation of individual cells is particularly useful when considering the cytotoxic effects of radiation, as the reaction of each agent to radiation is distinct. For example, the individual probabilities of mitosis, apoptosis and quiescence together determine the tumor regression, persistence or progression in response to a treatment.^35^

Agent-based modeling benefits from a wide range of pre-existing software, helping kick-start the model development while reducing the introduction of errors in the underlying model.^36^ These tools have two main approaches: to restrict cells to a fixed grid (on-lattice) or not (off-lattice). On-lattice models, like Cellular Automata, where each lattice site contains at most one cell, simplifies large-scale simulations,^37^ but they generally lack the ability to capture realistic cell shapes or intercellular forces.^32^ Cellular Potts Models, on the other hand, offer more realistic representations of cell shapes and growth patterns by allowing cells to occupy several lattice sites and having changes in the system governed by the minimization of the system's energy, useful for simulating amoeboid cell motion, cellular rearrangements, and internal cellular pressure among many other biological phenomena and properties.^38^ This model follows a formalism that is generally extended by including several terms in the Hamiltonian of the system^39^), but faces challenges such as lengthy computation times and difficulty in parameter sensitivity analysis due to some parameters lacking direct physical analogs.^40^

In contrast to lattice-based models, off-lattice models provide cells the freedom to move in space without the constraints of fixed grid locations, allowing for a more continuous exploration of mechanical effects on cell populations.^41^ This freedom facilitates more accurate simulations of cellular dynamics and interactions, as positions are tracked by their geometrical centers rather than predefined grid points.^37^ Among the off-lattice approaches, two center-based models, Overlapping Spheres and Voronoi Tessellation, offer distinctive advantages to simulating complex tumor environments. Overlapping Spheres models use spheres to represent individual cells or clusters, effectively simulating tumor growth and cellular interactions in three-dimensional space. As these spheres grow and overlap, they model physical forces such as pressure, adhesion, and repulsion among cells, crucial to understand tissue mechanics and tumor structuring.^42^ Conversely, Voronoi Tessellation involves computing the positions of cell centers and then determine the shape of each cell by maximizing its size relative to its neighbors. This tessellation forms a mosaic that realistically models the distribution and interaction of cells, reflecting both competition and cooperation within the tumor microenvironment.^43^ However, while providing detailed spatial resolution, these models face challenges in parameter sensitivity analysis. The computational capacity required for accurate simulations means that parameter adjustments can be time-consuming and require substantial computational resources, although parameter ranges can be effectively inferred from experimental data, which is not the case, in general, with Cellular Potts models^40^ (Table 1).

**Table 1.** Comparison of Common Modeling Approaches in Radiobiology, Outlining Their Core Advantages, Limitations, and Most Suitable use Cases.

	Model	Advanta es	Limitations	Best use case
	ODE	+ Capture essential biological dynamics + Fit well to experimental data	— Do not account for spatial differences — Choosing the correct model structure can be tric	Modeling average tumor growth trends or treatment responses in well-mixed, homogeneous populations; suitable for earlystage tumors or theoretical explorations.
On-lattice ABM	Cellular Automata	+ Allow largescale simulations + Represent each cell individually	— All changes are random and abrupt — Cells have fixed shape	Large-scale simulations of tumor growth with simplified rules and many cells; useful when computational speed is prioritized over detailed spatial or mechanical accuracy.
	Cellular Potts Model	+ Cells can take on any shape + Captures crawling or amoeboid cell motion	— Computationally intensive — Some parameters lack real-world analogs	Simulating individual cell shapes and motility in tissues; useful for studying phenomena like amoeboid movement, cell sorting, or cell-cell adhesion.
Off-lattice ABM	Overlapping Spheres	+ Tracks exact cell positions + Equations of motion are intuitive and permit easy extensions	— Can't accurately represent highly deformed cell shapes	Modeling mechanical interactions (eg, pressure, adhesion) among cells in 3D tumors; well-suited for studies on spatial structuring and tissue mechanics.
	Voronoi-Tessalation	+ Captures realistic, dynamic cell shapes + Can model complex tissue deformation	— Slow computations make sensitivity testing diffcult	Exploring cell packing, shape deformation, and competition/cooperation in dense tissue environments; best for realistic spatial distribution in tumors.

Hybrid modeling techniques represent a sophisticated integration of mechanical and biochemical modeling approaches, crucial for more accurately simulating the complex tumor microenvironment. These models combine ABM with reaction-diffusion partial differential equations (PDEs) to describe various effects introduced by the medium on the progression of the tumor, such as nutrient diffusion and metabolic waste production.^44^ The ability to simulate the diffusion and consumption of critical elements, like oxygen or nutrients, and the production of metabolic byproducts, allows researchers to observe how tumors adapt and respond dynamically to their environments. The inclusion of pro-angiogenic factors, matrix-degradation enzymes, and the diffusion of chemotherapeutic drugs can be modeled to study their effects on angiogenesis, invasion, metastasis, and treatment efficacy.^45–47^ Such hybrid models provide crucial insights into tumor behavior, offering a powerful tool to develop more effective therapeutic strategies and enhancing our understanding of how tumors interact with their microenvironment at a biochemical level.

### Irradiation Modeling

The integration of cell-growth models with irradiation simulation opens a broad array of possibilities to understand in detail, with high resolution, the radiobiological effects of radiotherapy. These models provide a framework for simulating the direct and indirect impact of radiation on DNA, which is the primary mechanism through which radiotherapy seeks to eradicate cancer cells. By incorporating the effects of radiation as an additional term in ODEs, these models can dynamically represent changes in tumor volume and viability in response to various dosing regimens. One commonly used approach is the LQ model, evaluating the biological impact of different radiation doses and fractionation schemes.^48^ However, the LQ model often falls short when predicting outcomes for higher radiation doses and does not adequately account for sub-lethal damage repair, which can significantly affect therapeutic efficacy.^12^ To address these limitations, current research is exploring the use of more sophisticated models that estimate ionizing radiation effects at the subcellular level. These allow for a better understanding of how clustered DNA damage and subsequent mutations and misrepairs contribute to cytotoxicity. These advanced models incorporate mechanisms such as DNA repair pathways and the stochastic nature of genetic damage, providing a more accurate prediction of radiation biological effects.

The integration of cell-proliferation models with advanced Monte Carlo (MC) simulation tools holds significant potential for improving our understanding and modeling of the biological effects of radiotherapy and enhancing the precision of cancer treatments. MC algorithms are capable of simulating complex particle interactions and, as such, are becoming an integral to some commercial radiotherapy treatment planning systems (TPS).^49,50^ These algorithms and models aid treatment planning and execution by applying fundamental radiobiological principles in clinical settings. Several MC tools are employed, each with specific strengths and shortcomings. Below is a comparison table detailing some advantages and potential weaknesses of some of the most widely used tools, as shown in Table 2.

**Table 2.** Overview of Some Widely Used Monte Carlo Simulation Tools in Radiobiology, Summarizing Their Focus Areas, key Strengths, and Known Limitations.

Feature	GEANT4	FLUKA	PENELOPE	MCNP
Developer	CERN/Geant4 Collaboration	CERN/INFN	NEA	LANL
Strengths	− Object-oriented, flexible design − Transparent code structure − Ecosystem of tools GATE/TOPAS	− Robustness across energy ranges − Clinically validated for high energy physics	− Advanced photon/electron simulation − Simplified user interface for e-/e+	− Gold standard for neutron transport − Broad particle support (37 types) – Advanced variance reduction
Focus	− General-purpose − Nuclear medicine − Proton therapy/imaging (via GATE/TOPAS)	− Beam-machine interactions − Radioprotection − Hadron therapy (HIT/CNAO)	− Low-energy photon/electron transport − Medical physics/dosimetry.	− Neutron transport − Reactor physics/shielding – Nuclear safeguards
Potential Weaknesses	− Steep learning curve.	− Limited lowenergy applications	− Computationally intensive for high energy e-/e+	− Export control restrictions.

Despite their widespread use, these MC tools face challenges in accurately modeling interactions at the DNA scale. This limitation arises from the condensed history techniques these tools employ, which compress multiple particle interactions into a single event, thereby neglecting crucial low-energy transfers essential for precise DNA damage modeling.^51^ Such oversimplifications can impact the accuracy of predicting how radiation affects cellular structures at the molecular level. To address these challenges, ongoing research focuses on enhancing the fidelity of simulations by improving the resolution at which these interactions are modeled. Efforts include developing algorithms capable of detailed energy transfer tracking and integrating these advancements into existing MC frameworks. This would allow for a more comprehensive understanding of radiobiological effects at the cellular and subcellular levels, crucial for designing future higher quality treatments, that are not only effective but also minimize potential side effects by, as much as possible, sparing healthy tissue.^52^ As these technological enhancements continue to evolve, they promise to refine the precision of radiotherapy, ensuring that cancer treatments are based on a detailed understanding of how radiation interacts with cellular DNA, thereby demonstrating a potential to optimize therapeutic outcomes.

## Applications of *in Silico* Modelling in Radiobiology

*In silico* models enable researchers and clinicians to overcome traditional experimental limitations and gain insight into the complex interactions within cellular and molecular systems. These models facilitate a detailed examination of how radiation affects biological tissues, offering new awareness, and enhancing our understanding of both the mechanisms of action and the potential of some proposed therapeutic interventions. In this section, we will explore various themes through which *in silico* models are employed to elucidate and innovate within the field of radiobiology. By simulating diverse biological processes and treatment responses, these models not only explain existing phenomena but also suggest potential strategies for optimizing some cancer treatments.

### Modelling Carcinogenesis

#### Cancer Stem Cells

The hypothesis of existence and presence of cancer stem cells (CSC) within tumors can play an important role in initiating and sustaining growth across a wide array of malignancies, as postulated by the CSC hypothesis.^53^ Despite their small number, CSCs have a large impact on tumor growth and treatment outcomes, making them a critical focus in cancer research. Utilizing the cellular Potts model, researchers have been able to study the emergent properties of CSC-driven tumor growth. This model has revealed that tumors with a small subset of CSCs exhibit increased heterogeneity and tend to adapt better to less optimal conditions, rather than only thriving in ideal growth environments. This adaptability results in higher overall fitness over time, leading to a more aggressive tumor phenotype that is better equipped to withstand and adapt to selection pressure such as therapeutic interventions.^54^ Such insights highlight the need for targeted therapies, that can effectively address the unique properties of CSCs and prevent them from driving tumor progression and resistance to treatments.

#### Cancer Vascularization

The role of *in silico* models in cancer research is underscored by their application in examining the transition from avascular to vascular tumor growth, a process for tumor progression towards more aggressive phenotypes. As solid tumors grow, cells at the center often become deprived of oxygen and nutrients, resulting in hypoxia. Severe or prolonged hypoxia may lead to necrosis, prompting hypoxic cells to drive vascularization. This process has been effectively simulated in studies such as that by Abbas Shirinfard et al, who modeled the secretion of pro-angiogenic factors by 3D spheroid cells when oxygen levels drop below a critical threshold.^47^ This model not only helps to understand how tumor-induced vasculature influences growth and morphology but also has the potential to assist in personalized medicine, by tailoring treatment strategies, both chemo and radiotherapy, based on tumor vasculature dynamics. Further insights into tumor microenvironments have been provided by Joshua A. Bull et al, who used an off-lattice model to simulate the differential pressures within a growing tumor, identifying distinct zones of proliferating and necrotic cells.^32^ Such detailed modeling helps delineate the spatial arrangement and state of cells within tumors, informing strategies for optimized drug delivery. For example, understanding enhanced interstitial convection within tumors, as discussed by R.K. Jain,^55^ can be important to improve the efficacy of macromolecule-based chemotherapies. These *in silico* models can, in the future, support radiotherapy planning by incorporating data on tumor architecture and vascularization, factors that would influence treatment outcomes.

#### Cell Cycle

The radiosensitivity of tumor cells is not constant, but varies throughout the cell cycle, influenced significantly by the cell's type and phenotype. This variability plays a role in radiotherapy, as treatment effectiveness can be influenced by the cell cycle phase at which cells are targeted. However, identifying phases of heightened or reduced sensitivity, which can differ across cell lines^26^ remains a challenge in optimizing clinical outcomes. *In silico* models of cell growth incorporate the dynamics of the cell cycle by simulating the duration of each phase and the checkpoints that regulate progression. These models adjust cell responses based on environmental conditions, such as nutrient availability and growth factors. For example, when nutrient levels or oxygen pressure drop below critical thresholds, cells may enter a quiescent state, remaining inactive until conditions improve. Prolonged adverse conditions can lead to cell necrosis.^56^ Furthermore, unlike normal cells, which halt proliferation under high contact interaction to maintain tissue homeostasis,^23^ cancer cells often lack this contact inhibition mechanism, continuing to progress through the cell cycle even under stressful conditions. This distinction is emphasized in models like those developed by Kempf et al, where cell-cycle progression is influenced by external conditions, specifically local glucose availability and interactions with neighboring cells mediated by integral pressure.^57^ Expanding these models, Jiang et al implemented a multiscale cellular Potts model that includes a protein regulatory network to realistically simulate the G1/S cell-cycle arrest. Their model divides the G1 phase into six stages, with progression through each one controlled by the local concentration of growth and inhibitory factors. This regulatory mechanism acts as an on/off switch, determining whether cells advance through the cell cycle or are halted.^46^ These *in silico* models have the potential to support radiotherapy research by simulating tumor responses based on the intrinsic cell cycle dynamics of cancer cells. Theoretically, if possible, aligning radiation doses with phases of maximum sensitivity could enhance treatment efficacy, but the complexity of patient biology and current limitations in radiobiological understanding mean that such models are still far from application in clinical decision-making.

#### Cell Proliferation and Clonal Evolution

Clonal selection and evolution can play key roles in the initiation and progress of neoplasms. The process starts when cells escape normal growth controls, gaining a proliferative advantage that enables their clonal expansion. As these clones undergo genetic instability, they give rise to variants; while most of them die out, some exhibit selective advantages, allowing their progeny to dominate until an even more favorable variant arises.^58^ A critical concern in radiotherapy is the potential for treatment to selectively kill only the most radiosensitive cells, inadvertently promoting the survival and dominance of more aggressive phenotypes. Computational models can offer powerful tools to examine such phenotype selections and their potential impact on tumor evolution and treatment outcomes. These models can simulate how certain traits—like the ability to induce angiogenesis, the presence of cancer stem cells (CSCs), or the propensity to metastasize—contribute to a tumor's success and adaptability under treatment pressures. Different models of cell phenotype evolution, often described as branching processes, enable the simulation of mutation accumulation over time.^59^ For instance, Anderson et al implemented an agent-based model (ABM) to track tumor evolution, considering each cell's life cycle, phenotype, and microenvironment^60^ This model highlights the significant clinical interest of genetic heterogeneity within tumors and the complex role of the microenvironment in modulating this heterogeneity.^61^ When comparing different evolutionary models, one approach follows a linear progression to increasingly aggressive phenotypes, known as the Vogelstein model, characterized by the sequential accumulation of specific oncogenes.^62^ Another approach involves random mutations among 100 preset phenotypes. Interestingly, the random mutation model showed that only a few phenotypes tend to dominate, often those with traits conducive to surviving in hypoxic conditions—traits like high proliferation rates, lack of cell-cell adhesion and low oxygen consumption.^60^ These findings underscore the challenges in targeting these adaptive, aggressive cancer cells in radiotherapy, highlighting the need for strategies that can anticipate and counteract these evolutionary dynamics due to selective pressure.

#### Immunogenic Effects of Radiotherapy

Beyond directly killing cancer cells through DNA and other damage, radiation can have a dual impact on cancer treatment. It enhances tumor cells’ visibility to the immune system, activating both innate and adaptive immunity to foster a targeted anti-tumor response.^25^ Cancer immunotherapy builds on this by stimulating the host immune system to recruit and activate cytotoxic cells specifically aimed at eliminating tumor cells. Numerous preclinical and clinical studies show that radiation can activate and strengthen the immune response to help control cancer. For instance, irradiation enhances the activity of CD8+ T lymphocytes, which are important for post-radiation tumor control.^63^ Radiation also modulates tumor cell surface markers, such as death receptors, tumor-associated antigens, and adhesion molecules, making tumor cells more susceptible to immune-mediated killing.^64^ Understanding the immune effects of radiotherapy and its synergy in immuno-oncology requires identifying optimal treatment schedules and tumor microenvironment conditions. Since extensive testing of dose-fractionation and tumor micro environmental factors is impractical in preclinical or clinical settings, advances in radiobiology and radiation-immune interactions offer the chance to explore these effects on the tumor microenvironment *in silico*.^65^ Reflecting this growing interest, Alfonso et al^66^ developed a three-dimensional ABM to simulate the dynamics of tumor–immune interactions under radiotherapy, capturing the balance between anti-tumor effector and pro-tumor suppressor immune phenotypes. Based on this framework, they introduced the “Radiation Immune Score” (iRIS), a data-driven metric designed to predict whether radiotherapy will induce favorable shifts in the tumor–immune ecosystem toward immune-mediated tumor elimination. The model was validated using data from 10 469 patient samples across 31 tumor types, and the iRIS was further shown to predict local control and overall survival in a cohort of non–small cell lung cancer patients treated with postoperative radiotherapy. Key features of the tumor microenvironment, including tumor cell proliferation and immune cell infiltration, are incorporated to stratify tumor–immune ecosystem into the three phases of cancer immunoediting: elimination, equilibrium, and escape. In their analysis, all clinical samples mapped to model-predicted compositions consistent with immune escape, supporting the model's ability to reflect clinically apparent disease states.

While the iRIS metric focuses on predicting local immune shifts within the tumor microenvironment to guide radiation dose adaptation, other models have addressed the systemic immunological consequences of treatment. Radiotherapy, beyond its local cytotoxic effects, can have immunosuppressive effects.^67^ It commonly induces radiation-induced lymphopenia—a depletion of circulating lymphocytes that is associated with poor outcomes in patients with solid tumors. Recognizing the immune system as a critical organ-at-risk, Jin et al^68^ developed a mathematical framework to model and predict radiation-induced lymphopenia. Their approach defines a five-compartment immune system—circulating blood, bone marrow, spleen, lymph nodes/vessels, and other lymphatic tissues—and simulates lymphocyte trafficking between these compartments. By accounting for radiation doses delivered to fixed anatomical sites and incorporating blood flow dynamics, the model captures dose–volume effects on the mobile lymphocyte pool. When combined with patient-specific radiosensitivity and lymphocyte regeneration capacity, this framework enables individualized prediction of radiation-induced lymphopenia severity, offering a valuable tool to minimize systemic immune suppression during radiotherapy.

However, while Jin et al's model addresses the immunosuppressive effects of radiotherapy, most computational efforts to date remain focused on tumor control and toxicity. As *Sung et al* noted,^67^ the broader immunological impact of radiotherapy—including its immunostimulatory potential—remains underexplored in treatment planning models. To address this gap, Sung et al developed a tumor–immune interaction model specifically for hepatocellular carcinoma, calibrated using circulating lymphocyte counts measured in patients during and after radiotherapy. The model includes both tumor and immune compartments and distinguishes between lymphocyte recruitment driven by tumor burden and that induced by antigen release from radiation-inactivated tumor cells. Crucially, it simulates how different fractionation regimens affect both lymphocyte dynamics and tumor control. Their findings suggest that hypofractionated schedules - delivering larger doses per fraction^69^ – can shorten the duration of lymphopenia while enhancing antigen-driven immune stimulation, creating more favorable conditions for subsequent immunotherapy. This model underscores the importance of balancing immune activation and suppression in radiotherapy design and provides a clinically grounded framework for optimizing fractionation in radio-immunotherapy protocols.

The combined effects of immunotherapy and radiotherapy were modeled by Sung et al,^70,71^ who developed a mathematical model to predict patient responses to radiotherapy with immune checkpoint inhibitors, specifically anti-CTLA-4 therapy. CTLA-4 is a crucial checkpoint in the cancer immunity cycle, and blocking it enhances T cell activation.^72^ The model divides cells into four compartments: irradiated tumor cells, dying tumor cells, non-irradiated tumor cells, and circulating lymphocytes. The model is then calibrated using data from CTLA-4 monotherapy clinical trials, incorporating a parameter that simulates the impact of CTLA-4 inhibition. Using this approach the model can predict patient responses to combined immunotherapy and radiotherapy. Results showed that radiation alone could control tumors locally, but the combination therapy was more effective for metastatic tumors. Additionally, results indicated that the immune checkpoint inhibitor (ICI) therapy should precede radiotherapy for optimal effect.^70^

Together, these models demonstrate the growing potential of *in silico* approaches to inform personalized radiotherapy strategies by capturing both local immune dynamics within the tumor microenvironment and systemic effects such as lymphopenia. By simulating the impact of various dose and fractionation schemes on immune activation and suppression, computational frameworks like iRIS and patient-calibrated immune models enable the exploration of treatment scenarios that would be impractical to test empirically. When rigorously validated, such models can support clinical trial design, optimize radio-immunotherapy protocols, and generate testable hypotheses about novel therapeutic combinations—all within a resource-efficient, data-driven framework.^65,70^

### Modelling Radiation Interaction

Monte Carlo track-structure (MCTS) codes, such as GEANT4-DNA, utilize advanced radiation transport models to provide intricate microscopic insights into the interactions between ionizing radiation and DNA structures. These simulations are key for integrating the complex geometry of DNA with the stochastic processes that govern radiation interactions and the subsequent chemical reactions leading to DNA damage.^73^ The extent and nature of DNA damage are significantly influenced by the radiation track structure and the organization of chromatin,^74^ as the spatial distribution of energy deposition directly impacts the type of DNA damage incurred. Variations in DNA density and arrangement, which differ among cell types and phases of the cell cycle, play a crucial role in these interactions and consequences.^75^ The models simulate DNA structure using a repeating sequence of nucleosomes arranged to form chromatin fibers. These kinds of models provide a visual context to understand how the organization of nucleosomes within the chromatin can influence the extent and type of radiation-induced DNA damage.

**Figure 1.**
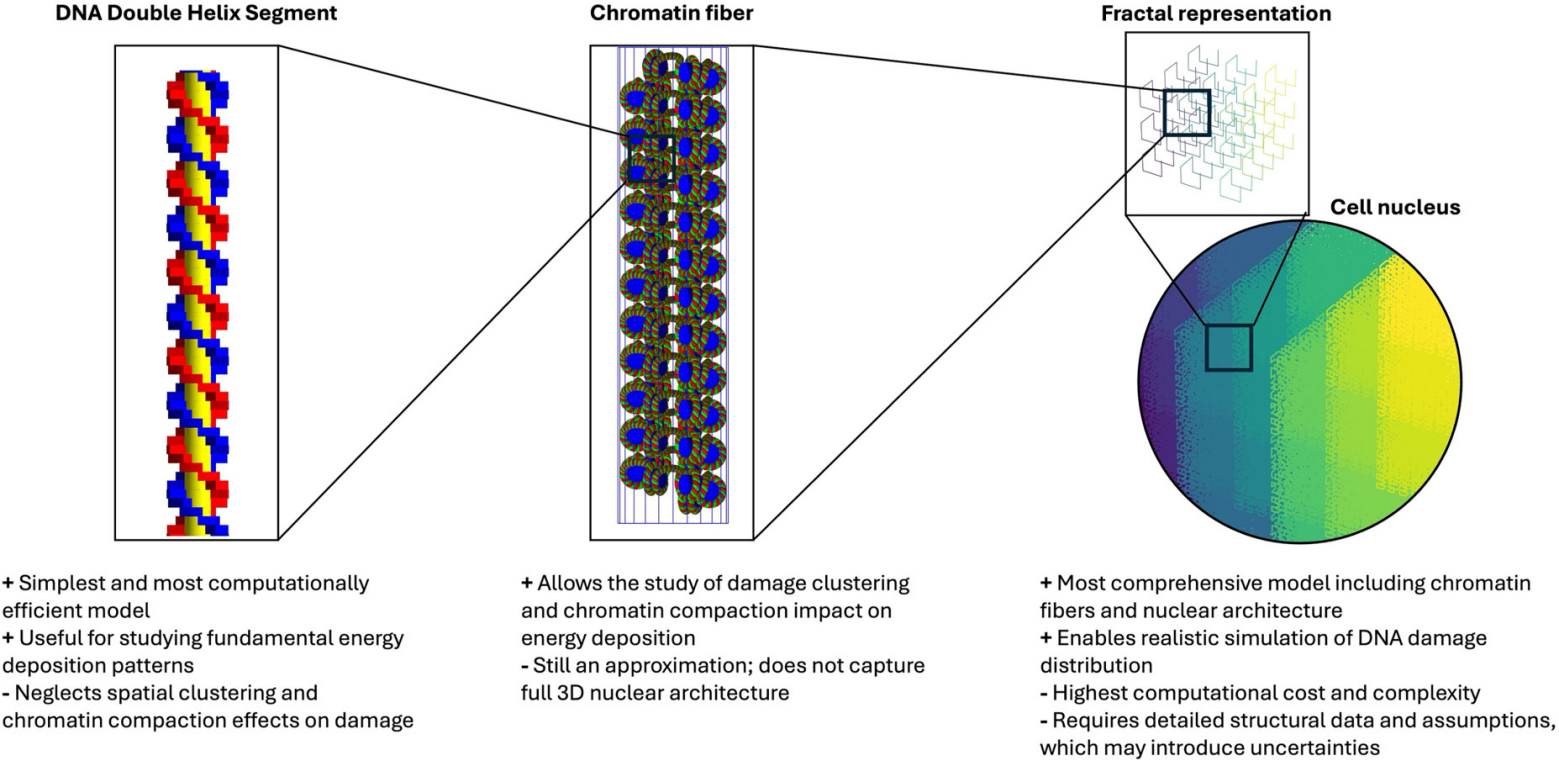
Progressive Levels of DNA Geometry Available in TOPAS-NBio to Study Radiation Effects—from Simple Linear Strands to Dense Chromatin Structures—Demonstrating the Trade-off Between Biological Realism and Computational Feasibility.

In radiotherapy modeling using MCTS codes, such as TOPAS-nBio^52^ (Figure 1) the geometric representation of DNA plays a critical role in simulating radiation interactions. The DNA fiber is modeled in various shapes—half-cylinder, cylinder sector, or spheres—which significantly influences the simulation outcomes. For instance, when irradiated with protons, the number of average strand breaks (SB) per Gy per base pair from direct damage varies significantly among these geometries. With the same LET, strand breaks can range from 10 SB in spheres to 50 SB in half-cylinders, with cylinder sectors showing intermediate values.^76^ This variation is attributed to the backbone volume of the models; the half-cylinder, lacking empty space between base pairs, exhibits a higher frequency of breaks. Interestingly, while the number of SB from direct damage remains relatively constant with increasing LET, indirect damage shows a decrease in strand breaks with increasing LET. This pattern is echoed in studies by Sakata et al who used a fractal-based DNA geometry with realistic base pair density. Their findings indicated that although the DSB increased with higher LET, SSB decreased as more breaks merged to form DSBs.^77^ The decrease in indirect damage strand breaks with increasing LET was linked to the high density of radiolytic products, leading to chemical neutralization rather than interactions with DNA.

The reactive nature of the hydroxyl radical, a key radiolytic product, makes it a primary agent in indirect DNA damage, often considered exclusively when assessing radiation impact on DNA.^78^ Such insights are crucial as they inform the selection and optimization of radiation types and dosages, aiming to maximize therapeutic efficacy while minimizing damage to healthy tissues.^20,79^ The exact structure of chromatin fibers remains an area of ongoing research, with significant implications in understanding the full extent and consequences of DNA damage during radiotherapy. While various models of chromatin structure have been proposed, Henthorn et al investigated three distinct models to explore how structural variations might affect radiation-induced strand breaks.^80^ The study aimed to determine if structural differences could lead to variations in the ratio of SSBs to DSBs when exposed to different types of radiation particles (protons and α-particles) and varying levels of LET. The findings revealed that despite the structural variations, there was little difference in the ratio of SSBs to DSBs across the models, suggesting that our current understanding of chromatin's influence on DNA damage may need refinement. This indicates that other factors may play a more significant role in determining radiation sensitivity and damage outcomes than previously thought. The solenoidal model of chromatin, as described by Henthorn et al has been effectively implemented in simulation tools such as TOPAS-nBio^52^ and constructed using DNAFabric.^81^ These tools allow for more precise simulations of DNA and chromatin interaction with radiation, providing researchers with the ability to explore and visualize how different chromatin configurations might influence DNA damage under various radiation conditions.

The GEANT4-DNA framework^82–85^ is extensively used for simulating the interactions between ionizing radiation and DNA, offering detailed insights into the molecular dynamics of radiation effects. This framework facilitates the inclusion of diverse reactions and reaction rates beyond those essential for simulating water radiolysis. For instance, in the study by Tang et al,^86^ the interaction of water radicals with DNA components, such as deoxyribose, phosphate, bases, and histones, was modeled within an anoxic environment. Although recognized by the authors as an unrealistic setting, this approach allowed to correlate simulated double-strand breaks (DSBs) with experimental γ-H2AX foci, a biomarker used to detect DSBs.^87^ The simulations achieved good agreement across three different x-ray irradiations with varying energies. The simulation of water radiolysis is done step-by-step in three stages. The first stage simulates the ionization of water molecules resulting from the interaction of the primary ionizing particles and their secondaries. Next are simulated the initial chemical species resulting from the decay of excited states or auto-ionization of water molecules, along with the thermalization of sub-excitation electrons. Finally, the chemical species diffuse and react with each other, producing new chemical species and reducing their initial number.^87^ This level of detail allows for a realistic simulation of the OER, but, for now, the medium where the simulations occur is limited to water with neutral pH and an ambient temperature of 25 °C.^88^ However, there is already work being done in this area, as shown in a study by Lai et al, where the OER was simulated using gMicroMC, a GPU-based MCTS.^89^ The authors found a good agreement between the experimental and simulated results, where for a low-LET x-ray beam incident in water with pO_2_ of around 14 mm Hg, the OER was 3.0, when compared to a completely hypoxic environment, where the OER plateaued. For proton beams with a LET of around 1.8 keV/μm, the maximum OER was 2.98. As the LET of the proton beam increased, the OER decreased to close to 2.65 for a LET of 19 keV/μm. Further studies for protons with higher LET would be interesting to analyze, as this value is still far from the proton beam LET that has the highest RBE. This value, at the low end, is around 30 keV/μm,^90^ but it should be noted that the most common value is assumed to be around 100–120 keV/μm as seen in,^4,74^ but this is something that should be discussed and studied for its validity, as proposed by B. Jones and M. A. Hill.^91^

#### Models of DNA Repair

Radiation primarily exerts its lethal effects on cells through DNA damage, which can be repaired, misrepaired, or left unrepaired. For cancer cells, which have lost faithful copy integrity,^20^ the goal of radiation therapy is to maximize damage while sparing healthy cells. Following radiation exposure, a damaged cell faces two potential fates: 1) it may sustain an unrepaired break leading to cell death, or 2) it may attempt to repair the damage. Although various types of DNA damage can lead to cell death, modeling efforts have predominantly focused on DSBs due to their strong correlation with cell lethality.^17^ There are three primary mechanisms for DSB repair: Nonhomologous End Joining (NHEJ), Homologous Recombination (HR), and Microhomology-Mediated End Joining (MMEJ).^15^ These mechanisms differ in the accurateness of repair, which is mainly due to the presence of a homologous DNA template,^92^ which in turn is dependent on the phase of the cell cycle.

NHEJ, which can operate throughout the cell cycle, is the predominant repair mechanism in normal cells, providing a quick repair response to DSBs. HR, available only during the late S and G2 phases, offers a more precise repair of complex DNA damage. In instances where NHEJ or HR is inhibited, cells may resort to MMEJ as an alternative; however, this method is less accurate due to its lack of reliance on sequence homology.^93^ Both NHEJ and MMEJ are error-prone processes that can lead to genetic alterations, potentially resulting in cell death.^20,94^ Due to its speed and prevalence across all cell cycle phases, NHEJ is the most frequently simulated DNA repair mechanism in radiobiological models.^95^ One example of the inclusion in simulation of NHEJ repair can be found on the DaMaRiS Framework.

The DaMaRiS framework, developed at the University of Manchester, offers a sophisticated model for simulating the motion and reaction of DNA DSB repair proteins.^96^ This computational scheme begins by reading the locations of DSBs from a Standard DNA Damage file,^97^ where each DSB is disconnected into two separate ends. The repair process for each end is meticulously tracked, starting with the recruitment of Ku70/80 repair proteins, followed by resection initiation, processing, ligation, and the final repair stages. This system has demonstrated a good agreement with existing models of DNA repair, supporting its validity and utility in simulating complex biological repair processes. However, some experimental behaviors remain elusive, particularly in scenarios modeled in the framework that involve either linear pathways, with distinct fast and slow repair routes, or intertwined pathways.^96^ Further development by Ingram et al has expanded the framework's capabilities to include HR alongside NHEJ for DSB repair. This expansion allows the model to explore various repair pathway scenarios: a “NHEJ first” approach where HR is only engaged if NHEJ fails, and a “competition” approach where the pathway choice is dictated by the kinetics of protein recruitment. Should a repair attempt fail, the DSB end becomes available again for repair by either mechanism.^95^ Comparative analysis against experimental results, including studies on cell types deficient in XLF and Lig4 - key proteins in the NHEJ pathway^98^ - revealed that pathways based solely on competition were less consistent with experimental data. This suggests that even subtle variations in the choice of repair mechanism can significantly influence the overall repair kinetics. The distinctiveness of the DaMaRiS framework lies in its explicit modeling of protein recruitment dynamics during DNA repair, offering a detailed, mechanistic view, of how repair proteins interact and influence the repair process. This level of detail provides important insights into the cellular machinery of DNA repair, potentially guiding the development of therapeutic approaches that can manipulate these pathways to enhance treatment efficacy and/or reduce adverse effects.

Adopting a distinct methodology, McMahon et al developed a model that simulates the rate of DNA repair and misrepair, calculating the probability of these events leading to cell death or significant genetic alterations. Central to this model is its ability to predict key biological endpoints—such as DNA repair kinetics and cell survival—by adjusting a universal set of parameters applicable across all cell types.^99^ This model, known as the Mechanistic DNA Repair and Survival Model (Medras), was initially designed to address the repair processes associated with sparsely ionizing radiation, assuming a uniform distribution of damage.^93^ Medras has since been expanded to encompass the temporal evolution of damage and to include the effects of high-LET radiation. This advancement allows the model to integrate damage data directly from Standard DNA Damage files, enhancing its accuracy and scope.^94^ The model comprehensively covers all known DNA repair mechanisms and can simulate the distribution of misrepair events and the types of chromosomal aberrations that may result. McMahon et al have demonstrated a strong correlation between the simulated repair events and experimental assays across various cell lines, types of radiation particles and irradiation conditions. This innovative approach not only enhances our understanding of radiation-induced DNA damage and repair but also holds significant potential to refine mechanistic predictions of radiation sensitivity. Such capabilities can be instrumental in developing new tools for some level of treatment personalization, ultimately contributing to more tailored and effective radiation therapy strategies.

## Challenges and Limitations

The significant advancements in *in silico* modeling are largely propelled by the substantial improvements in computational power now accessible to the scientific community. While these advancements have expanded the scope and potential of *in silico* modeling, the challenge remains to develop models that are well suited to real-world data.

A primary limitation in *in silico* modeling is the scarcity and variability of clinical datasets, which are often inadequate for training robust models and may exhibit considerable heterogeneity across experimental conditions. This issue is particularly pronounced in radiation biology, where both systematic and statistical uncertainties associated with biological endpoints are compounded by errors in the physical determination of the delivered dose.^100^ Addressing these challenges requires a high degree of interdisciplinary collaboration among clinicians, experimentalists, and theoretical scientists. Such collaboration should prioritize the development of standardized protocols to optimize the acquisition of high-quality, relevant data for model calibration and validation. These efforts are essential for constructing mechanistically grounded models that advance the understanding of radiobiological mechanisms while supporting their integration into clinical practice. Furthermore, standardizing experimental design and dosimetry reporting across research groups could reduce uncertainties related to dose administration, tissue-specific responses, and cellular heterogeneity,^101^ thereby enhancing the reproducibility and predictive accuracy of computational models. Guidelines for essential information to report in radiobiological experiments are detailed in Hill et al^100^ while Desrosiers et al^102^ provide recommendations for reporting experiments used in model calibration.

Additionally, as models grow increasingly complex with the integration of artificial intelligence and machine learning into clinical practice, they face interpretability issues. These advanced models often lack transparency, complicating the verification and understanding of their decision-making processes.^31^ Non-interpretable models—often referred to as “black-box” approaches—frequently outperform traditional, more interpretable techniques such as logistic regression and decision trees in predictive tasks.^103^ However, the opaque nature of these models poses significant limitations for clinical use. Clinical decision-making depends not only on predictive accuracy but also on an informed understanding of how patient-specific factors, radiation response, and model outputs interact. Improving interpretability and transparency helps clinicians better understand and anticipate model behavior, an essential step toward improving robustness, generalizability, and clinical trust.^104^ These come with major challenges, as, first of all, these algorithms need to be extensively tested for accuracy before clinical implementation, requiring costly and time-consuming testing.^105^ Moreover, the development and deployment of complex machine learning models, particularly deep learning architectures, typically require substantial computational resources, including high-performance graphical processing units and algorithmic optimization.^103,105^ These AI models typically required a large and high-quality dataset to train, as these factors heavily influence the model performance.^106^ Current clinical practice generates discrete, quantitative and structured patient-specific data, already in an electronic infrastructure within radiation oncology, that is well positioned for use in the training of these models. However, existing data are housed across different platforms, at multiple institutions and is often not stored in a standardized manner.^107^ While these technological demands do not inherently prevent clinical integration, they can represent significant practical barriers to broader adoption and scalability. However, the landscape is changing with technological advances in imaging. For instance, the integration of magnetic resonance imaging within radiation delivery devices promises to revolutionize data collection.^108,109^ These advancements are expected to provide a cost-effective mean to obtain more comprehensive data, thereby enabling the calibration and validation of more detailed and accurate mathematical models. Nonetheless, potential clinical application of these models must consider several the need of extra resources that could make the integration of *in silico* techniques difficult. There are costs associated with acquiring the necessary machines, training specialized personnel to work with these models, computational infrastructure, and potentially the costs of commercial software algorithms.^108^ Even in high-income countries, the number of radiotherapy facilities and trained staff is insufficient, limiting access to advanced treatments. In low-income countries, these challenges are even more pronounced, with significant gaps in infrastructure, a shortage of trained professionals and limited access to advanced technologies.^110^ As a result, the implementation of cutting-edge radiotherapy techniques and computational models is often not feasible, further widening the disparity in cancer care between high- and low-resource countries.

## Future Directions and Emerging Trends

*In silico* models have evolved from an innovative yet skeptically received concept in radiobiological research to a potentially valuable tool in a future clinician's arsenal. This transformation has been fueled by the exponential growth in computing power and the establishment of specialized teams dedicated to developing models and tools that incorporate not only the details of physics processes and chemical reactions, but also facets of artificial intelligence, including machine learning and deep learning.^111^

An illustration of the progress and direction in this field can be observed through the ongoing advancements and applications of artificial intelligence. Artificial intelligence is fast becoming an important component of radiotherapy, covering aspects such as medical imaging analysis, treatment planning prediction, and the simulation and verification of radiotherapy delivery dose.^112^ Recently, deep learning has emerged as a valuable tool, especially noted for its ability to predict patient-specific optimal dose distributions, thereby enhancing treatment planning quality. An innovative approach in this domain uses reinforcement learning (RL) and its advanced variant, deep reinforcement learning, which merges RL with cutting-edge deep learning advancements. Here, a computer agent trained to make autonomous decisions based on environmental observations operates the TPS. This virtual treatment planner replaces human planners, setting and adjusting dosimetric constraints to create high-quality plans.^113^ This methodology has the potential to enhance real-time TPSs for intensity modulated radiation therapy, by reducing the need for repetitive optimization and facilitating near real-time planning. It begins with fluence map prediction, followed by fine-tuning for specific cases like breast cancer, significantly cutting traditional planning times. Continued research has even allowed for fluence map prediction without an intermediate dose prediction step, streamlining the process further.^114^ These are just some examples of tools that can benefit greatly from the new era in technological innovation that we are currently on; developments in quantum computing and robust cloud-based systems paired with the rapid evolution of large language models are creating new opportunities and avenues for personalized medicine. Quantum computing, with its superior processing speeds, has the potential to enhance, in special applications, the capability for predictive healthcare, despite its high costs and complex maintenance. Leveraging cloud-based resources, like Amazon Braket, can alleviate these challenges by providing scalable quantum processing.^115^ Employing a hybrid approach, where classical systems handle initial setups and quantum computers undertake complex simulations, could significantly boost both the efficiency and accuracy of medical simulations.^116^ This synergy between advancements in *in silico* models, computational power, and novel techniques applied in a clinical setting may offer meaningful improvements in radiobiology, supporting more detailed and dynamic modeling of complex biological processes.

## Clinical Implications and Translational Perspectives

Over the past decades, radiation therapy has seen significant advancements in technology and treatment techniques. This progress is partly attributable to the increased capabilities of commercial radiotherapy TPS, which play a crucial role in planning, optimizing, and assessing patient treatments, ultimately determining the quality and efficacy of modern radiation therapy.^117^ The leading commercial TPSs include RayStation (RaySearch Laboratories, Stockholm, Sweden) and Eclipse (Varian Medical Systems, Palo Alto, CA, USA). These systems employ a range of dose calculation algorithms, from simpler homogeneous methods to more advanced convolution/superposition algorithms. However, MC methods offer the highest accuracy, particularly in modeling dose distributions within heterogeneous materials.^118^ Historically, MC algorithms were confined to specialized research software, limiting their clinical application. However, recent development of toolkits has made general-purpose MC codes more accessible to researchers and clinical staff in medical physics.^119^ MC codes are considered the most accurate dose calculation engines, simulating particle transport based on fundamental physical interactions. While current TPS have advanced significantly, *in silico* simulations offer immense potential as a complementary tool in radiotherapy planning, providing valuable support for tumor delineation and treatment optimization. Driven by significant advancements in computational technologies, these models can contribute to the development of more personalized treatments and to offer additional support to clinical decision-making processes. Datasets growth, in both size and complexity, employing high-throughput molecular and imaging-based methods, has increasingly enabled outcomes that closely correlate with clinical results.^120^ One of the most important applications of *in silico* models in clinical settings is their use in virtual clinical trials. Virtual clinical trials employ digital replicas of patients and simulate devices to mimic medical examinations and procedures, aiming to produce outcomes equivalent to those of traditional trials but without the associated costs, ethical concerns and time demands. For example, these trials can simulate x-ray breast projections or analyze glandular dose distributions within digital breast phantoms, significantly aiding scanner design optimization and protocol evaluation, while avoiding radiation exposure to real patients.^121^ Further advancing these applications, Schiavo et al have integrated a novel model of tumor vasculature and oxygenation into a virtual clinical trial framework for dose painting in stereotactic body radiotherapy. This innovative approach addresses the limitations of current imaging systems by simulating radiobiological responses to hypoxia, which is relevant for customizing treatments. By enhancing reoxygenation strategies and fighting radiation resistance in hypoxic tumors, this model opens new avenues for improving therapeutic outcomes in challenging clinical scenarios.^122^ Models that combine anatomical predictions with adapted treatment plans, such as adaptive radiotherapy, also enable the implementation of training strategies by utilizing prior knowledge, such as images and segmentations from the original treatment planning.^123^ These strategies could significantly improve treatment efficacy by dynamically adapting to changes in tumor volume.^124,125^ Additionally, adaptive approaches can be optimized through mathematical models that simulate individual patient tumors, predicting responses to different treatment modalities. These models can serve as dynamic biomarkers, allowing clinicians to tailor treatments more precisely to the specific characteristics of each patient's tumor.^126^ As the field advances, integrating these predictive models into clinical practice could improve cancer treatment by increasing personalization and then be more effective

**Figure 2.**
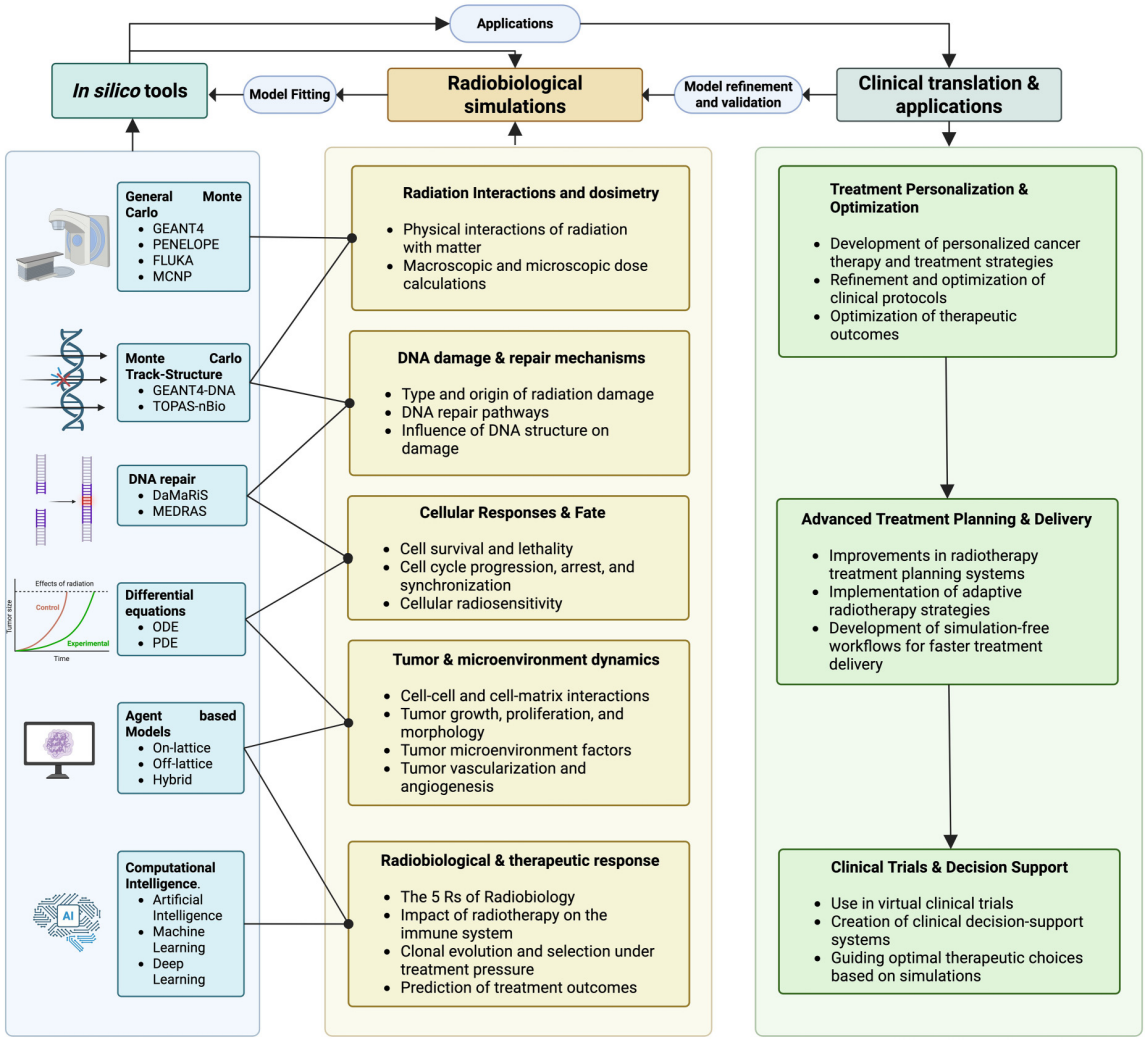
Conceptual Map Linking In Silico Modeling Tools To Radiobiological Processes And Clinical Applications, Highlighting Iterative Data-Driven Refinement.

*Source:* Created in BioRender. Azevedo T (2025) https://BioRender.com/j687jck

*In silico* models have the potential to enhance clinical decision-support systems by providing additional insights for treatment planning and facilitating post-treatment analysis, which may be used in the iterative refinement of these models*.* These systems expertly balance the benefits of treatments, such as tumor control and patient survival, against potential drawbacks, like toxic effects and high financial costs. By doing so, they provide physicians and patients with valuable insights to make informed decisions.^120^ Looking ahead, future enhancements of these decision systems are set to include comparative analyses of different therapeutic modalities, such as photon versus proton therapy. Additionally, integrating genetic information into these models aims to further personalize treatment strategies, tailoring interventions to the unique genetic profiles of individuals.^127^ *In silico* techniques have also been important in the development of simulation-free workflows, which significantly accelerate the delivery of treatments, like whole brain radiation therapy, without compromising the plan quality. These innovative approaches have been clinically validated for safety and efficacy, enabling faster treatment planning and delivery. As a result, patients benefit from more timely interventions and better preservation of cognitive functions—a critical consideration in whole brain treatments.^128^ The ongoing evolution of *in silico* models may further integrate these tools into clinical workflows, supporting patient care and treatment planning, as shown in Figure 2. With advancements in computational power, algorithmic development and interdisciplinary collaboration, these models have the potential to support personalized medicine and improve clinical outcomes.

## Conclusion

Substantial progress has been made in various aspects of *in silico* modeling, advancing both our understanding and the path towards the application of these tools in clinical settings. Cell growth models effectively capture cellular mutations and interactions with the microenvironment, including processes like cell proliferation and invasion, cell-cycle progression and arrest. These models translate complex biological processes into detailed simulations, such as those enabled by MC techniques, which track individual particles within the cell nucleus and simulate, for instance, the production of radicals from the water ionization. Such capabilities can help in guiding personalized therapy approaches, which would allow for treatment plans that maximize tumor damage while minimizing harm to healthy tissue. The spatial and temporal scales accessible through MCTS surpass what can be achieved experimentally, opening possibilities for exploring hidden variables that potentially influence treatment outcomes. Moreover, enhancing the resolution of radiation dose distributions in simulations using all-purpose MCTS simulations, like GEANT4-DNA, instead of relying on analytic expressions suitable only for controlled experimental settings, could significantly improve the accuracy and efficacy of testing of novel treatment techniques.

Currently, integrated toolkits that combine cell growth and irradiation models are scarce, with RADCELL standing out as a notable example. This toolkit facilitates the transfer of tumor information from CompuCell3D (CC3D)^129^ to GEANT4-DNA, subsequently feeding back data on cell dose and DNA damage to CC3D to update cell properties.^130^ For those exploring MCTS, a comprehensive comparison of various tools is available in,^75^ which highlights GEANT4-DNA as one of the most complete and widely utilized, corroborated by its frequent citation in scholarly articles. However, it is crucial to note that GEANT4-DNA is highly specialized, primarily targeting DNA constituents and liquid water under very specific conditions. Despite this specialization, the availability of GEANT4 wrapper tools, as TOPAS-nBio and GATE, has facilitated the application of GEANT4's complex physical models across diverse applications. TOPAS-nBio is geared towards radiobiological experiments, while GATE is optimized for radiotherapy and imaging. Looking forward, these tools offer two promising pathways to advance clinical and research applications. In research, the most suitable cell growth model for a given experiment can be chosen, with a coupled toolkit similar to RADCELL transferring data to TOPAS-nBio for radiobiological simulation. Clinically, patient imaging data from modalities like CT and PET can be directly input into GATE to refine treatment plans. As the field of nanodosimetry matures, these integrated approaches are expected to deepen our understanding of radiobiological effects at the cellular level, with the high potential to drive more effective treatment plans and, consequently, improved patient outcomes. The integration of advanced simulation tools in clinical applications, even if in its infancy but with a high potential of expansion, represents a promising step towards more personalized medicine, where treatment strategies can be adjusted to better reflect the unique biological characteristics of each patient's condition.

## Abbreviations

ABM: agent based models
CSC: cancer stem cells
DSB: double strand break
HR: homologous recombination
LET: linear energy transfer
LQ: linear quadratic
MC: Monte Carlo
MCTS: Monte Carlo track structure
MMEJ: microhomology-mediated end joining
NHEJ: nonhomologous end joining
ODE: ordinary differential equation
PDE: partial differential evolution
ROS: reactive oxygen species
SB: strand break
SSB: single strand break
TPS: treatment planning system
